# Supplementary material for: The Optimization of the Hot Water Extraction of the Polysaccharide-Rich Fraction from Agaricus bisporus
Source: Molecules. 2024 Oct 9;29(19):4783. doi: 10.3390/molecules29194783 (PMC11478120; doi:10.3390/molecules29194783)
Supplement: Supplementary file 1 [file molecules-29-04783-s001.zip › molecules-3163143-supplementary.pdf]

Optimization of polysaccharide rich fraction hot water extrac-tion of Agaricus bisporus.

Aya Samy Ewesys Khalil, Marcin Lukasiewicz

Supplementary materials

TCC model

Table S1. Model Summary

S	R^2	Adjusted R^2	Predictive R^2
7.623	0.915	0.904	0.889

Table S2. ANOVA Table

Source	Sum of squares	df	Mean square	F	p
Model	19761.061	9	2195.673	37.780	< .001
Linear terms	936.527	3			
Time	356.839	1	356.839	6.140	0.015
Temperature	333.210	1	333.210	5.733	0.019
Liquid/solid Ratio	246.477	1	246.477	4.241	0.043
Squared terms	11573.136	3			
$Time^2$	1286.551	1	1286.551	22.137	< .001
$Temperature^2$	1152.208	1	1152.208	19.826	< .001
Liquid/solid $Ratio^2$	9134.377	1	9134.377	157.172	< .001
Interaction terms	7251.398	3			
Time·Temperature	5459.563	1	5459.563	93.941	< .001
Time·Liquid/solid Ratio	31.556	1	31.556	0.543	0.464
Temperature· Liquid/solid Ratio	1760.279	1	1760.279	30.288	< .001
Error	4300.676	74	58.117		
Total	24061.737	83			

Table S3. Coded coefficients

Alias	Term	Effect	Coefficient	Standard error	t	p	VIF
(Intercept)	(Intercept)		172.180	2.201	78.239	< .001	
β_1	Time	11.783	5.891	1.100	5.354	< .001	1.000
β_2	Temperature	44.536	22.268	1.100	20.237	< .001	1.000
β_3	Liquid/solid Ratio	17.613	8.807	1.100	8.003	< .001	1.000
β_{11}	$Time^2$	-16.372	-8.186	1.740	-4.705	< .001	
β_{22}	$Temperature^2$	-15.493	-7.747	1.740	-4.453	< .001	
β_{33}	Liquid/solid $Ratio^2$	-43.623	-21.812	1.740	-12.537	< .001	
β_{12}	Time·Temperature	30.165	15.082	1.556	9.692	< .001	1.000
β_{13}	Time·Liquid/solid Ratio	-2.293	-1.147	1.556	-0.737	0.464	1.000
β_{23}	Temperature· Liquid/solid Ratio	17.128	8.564	1.556	5.503	< .001	1.000

RS model

Table S4. Model Summary

S	R^2	Adjusted R^2	Predictive R^2
0.045	0.957	0.952	0.945

Table S5. ANOVA Table

Source	Sum of squares	df	Mean square	F	p
Model	0.677	9	0.075	36.959	< .001
Linear terms	0.082	3			
Time	0.005	1	0.005	2.306	0.133
Temperature	1.775×10^{-4}	1	1.775×10^{-4}	0.087	0.768
Liquid/solid Ratio	0.077	1	0.077	37.868	< .001
Squared terms	0.524	3			
$Time^2$	0.004	1	0.004	2.095	0.152
$Temperature^2$	1.220×10^{-4}	1	1.220×10^{-4}	0.060	0.807
Liquid/solid $Ratio^2$	0.519	1	0.519	255.379	< .001
Interaction terms	0.071	3			
Time·Temperature	0.029	1	0.029	14.352	< .001
Time·Liquid/solid Ratio	1.602×10^{-4}	1	1.602×10^{-4}	0.079	0.780
Temperature· Liquid/solid Ratio	0.042	1	0.042	20.404	< .001
Error	0.151	74	0.002		
Total	0.827	83			

Table S6. Coded coefficients

Alias	Term	Effect	Coefficient	Standard error	t	p	VIF
(Intercept)	(Intercept)		0.824	0.013	63.318	< .001	
β_1	Time	-0.011	-0.005	0.007	-0.823	0.413	1.000
β_2	Temperature	0.093	0.046	0.007	7.140	< .001	1.000
β_3	Liquid/solid Ratio	-0.465	-0.232	0.007	-35.691	< .001	1.000
β_{11}	Time^2	-0.030	-0.015	0.010	-1.447	0.152	
β_{22}	Temperature^2	0.005	0.003	0.010	0.245	0.807	
β_{33}	Liquid/solid Ratio^2	0.329	0.164	0.010	15.981	< .001	
β_{12}	Time·Temperature	0.070	0.035	0.009	3.788	< .001	1.000
β_{13}	Time·Liquid/solid Ratio	0.005	0.003	0.009	0.281	0.780	1.000
β_{23}	Temperature· Liquid/solid Ratio	-0.083	-0.042	0.009	-4.517	< .001	1.000

DDPH model

Table S7. Model Summary

S	R^2	Adjusted R^2	Predictive R^2
0.842	0.661	0.620	0.559

Table S8. ANOVA Table

Source	Sum of squares	df	Mean square	F	p
Model	73.591	9	8.177	11.546	< .001
<u>Linear terms</u>	29.445	3			
Time	4.886	1	4.886	6.898	0.010
Temperature	23.948	1	23.948	33.814	< .001
Liquid/solid Ratio	0.611	1	0.611	0.863	0.356
<u>Squared terms</u>	35.354	3			
Time^2	12.722	1	12.722	17.964	< .001
Temperature^2	22.368	1	22.368	31.584	< .001
Liquid/solid Ratio^2	0.263	1	0.263	0.372	0.544
<u>Interaction terms</u>	8.792	3			
Time·Temperature	0.041	1	0.041	0.058	0.810
Time·Liquid/solid Ratio	7.862	1	7.862	11.100	0.001
Temperature· Liquid/solid Ratio	0.889	1	0.889	1.256	0.266

Source	Sum of squares	df	Mean square	F	p
Error	52.408	74	0.708		
Total	125.999	83			

Table S9 Coded Coeffients

Alias	Term	Effect	Coefficient	Standard error	t	p	VIF
(Intercept)	(Intercept)		0.520	0.243	2.142	0.035	
β_1	Time	-0.926	-0.463	0.121	-3.811	< .001	1.000
β_2	Temperature	-1.888	-0.944	0.121	-7.772	< .001	1.000
β_3	Liquid/solid Ratio	0.765	0.382	0.121	3.147	0.002	1.000
β_{11}	Time^2	1.628	0.814	0.192	4.238	< .001	
β_{22}	Temperature^2	2.159	1.079	0.192	5.620	< .001	
β_{33}	Liquid/solid Ratio^2	-0.234	-0.117	0.192	-0.610	0.544	
β_{12}	Time·Temperature	-0.083	-0.042	0.172	-0.242	0.810	1.000
β_{13}	Time·Liquid/solid Ratio	-1.145	-0.572	0.172	-3.332	0.001	1.000
β_{23}	Temperature· Liquid/solid Ratio	0.385	0.193	0.172	1.121	0.266	1.000

H_2O_2 model

Table S10. Model Summary

S	R^2	Adjusted R^2	Predictive R^2
6.381	0.485	0.422	0.328

Table S11. ANOVA Table

Source	Sum of squares	df	Mean square	F	p
Model	2040.112	9	226.679	5.567	< .001
Linear terms	628.373	3			
Time	350.526	1	350.526	8.608	0.004
Temperature	88.063	1	88.063	2.163	0.146
Liquid/solid Ratio	189.785	1	189.785	4.661	0.034
Squared terms	265.102	3			
$Time^2$	0.247	1	0.247	0.006	0.938
$Temperature^2$	240.267	1	240.267	5.901	0.018
Liquid/solid $Ratio^2$	24.589	1	24.589	0.604	0.440
Interaction terms	1146.636	3			
Time·Temperature	1002.851	1	1002.851	24.629	< .001
Time·Liquid/solid Ratio	100.369	1	100.369	2.465	0.121
Temperature· Liquid/solid Ratio	43.417	1	43.417	1.066	0.305
Error	3013.194	74	40.719		
Total	5053.306	83			

Table S12. Coded Coeffients

Alias	Term	Effect	Coefficient	Standard error	t	p	VIF
(Intercept)	(Intercept)		74.717	1.842	40.561	< .001	
β_1	Time	3.917	1.959	0.921	2.127	0.037	1.000
β_2	Temperature	3.827	1.913	0.921	2.077	0.041	1.000
β_3	Liquid/solid Ratio	9.448	4.724	0.921	5.129	< .001	1.000
β_{11}	$Time^2$	0.227	0.113	1.456	0.078	0.938	
β_{22}	$Temperature^2$	-7.075	-3.537	1.456	-2.429	0.018	
β_{33}	Liquid/solid $Ratio^2$	-2.263	-1.132	1.456	-0.777	0.440	
β_{12}	Time·Temperature	12.928	6.464	1.303	4.963	< .001	1.000
β_{13}	Time·Liquid/solid Ratio	-4.090	-2.045	1.303	-1.570	0.121	1.000
β_{23}	Temperature· Liquid/solid Ratio	-2.690	-1.345	1.303	-1.033	0.305	1.000

ABTS model

Table S13. Model Summary

S	R^2	Adjusted R^2	Predictive R^2
4.211	0.937	0.930	0.918

Table S14. ANOVA Table

Source	Sum of squares	df	Mean square	F	p
Model	1140.414	9	126.713	7.147	< .001
Linear terms	304.285	3			
Time	157.597	1	157.597	8.889	0.004
Temperature	113.791	1	113.791	6.418	0.013
Liquid/solid Ratio	32.897	1	32.897	1.855	0.177
Squared terms	339.548	3			
$Time^2$	105.356	1	105.356	5.942	0.017
$Temperature^2$	111.092	1	111.092	6.266	0.015
Liquid/solid $Ratio^2$	123.100	1	123.100	6.943	0.010
Interaction terms	496.581	3			
Time·Temperature	91.924	1	91.924	5.185	0.026
Time·Liquid/solid Ratio	106.724	1	106.724	6.019	0.017
Temperature· Liquid/solid Ratio	297.933	1	297.933	16.804	< .001
Error	1312.036	74	17.730		
Total	2452.450	83			

Table S15. Coded Coefficients

Alias	Term	Effect	Coefficient	Standard error	t	p	VIF
(Intercept)	(Intercept)		70.303	1.216	57.838	< .001	
β_1	Time	14.774	7.387	0.608	12.154	< .001	1.000
β_2	Temperature	4.950	2.475	0.608	4.072	< .001	1.000
β_3	Liquid/solid Ratio	-36.286	-18.143	0.608	-29.852	< .001	1.000
β_{11}	$Time^2$	4.685	2.342	0.961	2.438	0.017	
β_{22}	$Temperature^2$	-4.811	-2.405	0.961	-2.503	0.015	
β_{33}	Liquid/solid $Ratio^2$	-5.064	-2.532	0.961	-2.635	0.010	
β_{12}	Time·Temperature	3.914	1.957	0.860	2.277	0.026	1.000
β_{13}	Time·Liquid/solid Ratio	4.217	2.109	0.860	2.453	0.017	1.000
β_{23}	Temperature· Liquid/solid Ratio	-7.047	-3.523	0.860	-4.099	< .001	1.000
